# Recent Advances in the Preparation and Application of Biochar Derived from Lignocellulosic Biomass: A Mini Review

**DOI:** 10.3390/polym16060851

**Published:** 2024-03-20

**Authors:** Kanglei Wang, Javier Remón, Zhicheng Jiang, Wei Ding

**Affiliations:** 1 China Leather and Footwear Research Institute Co., Ltd., Beijing 100015, China; wkljy1226@163.com; 2 Thermochemical Processes Group, Aragón Institute for Engineering Research (I3A), University of Zaragoza, C/Mariano Esquillor s/n, 50018 Zaragoza, Spain; jrn@unizar.es; 3 College of Biomass Science and Engineering, Sichuan University, Chengdu 610065, China; zhichengjiang@scu.edu.cn; 4 College of New Materials and Chemical Engineering, Beijing Institute of Petrochemical Technology, Beijing 102617, China

**Keywords:** lignocellulosic biomass, conversion, biochar, carbonization, activation, applications

## Abstract

With the rapid growth in the global population and the accelerating pace of urbanization, researching and developing novel strategies for biomass utilization is significant due to its potential for use in renewable energy, climate change mitigation, waste management, and sustainable agriculture. In this environmental context, this review discusses the recent advances in biomass conversion technologies for biochar production, including the first carbonization process and the subsequent activation methods of the biochar derived from lignocellulosic biomass (LBC). Parallel to this, this review deals with other essential parameters in biochar production, such as feedstock types, reaction environments, and operating conditions in the pyrolysis process, to determine the production and composition of LBC. Moreover, the wide-ranging applications of LBC in areas such as adsorption, catalysts, and energy storage are discussed, offering sustainable and environmentally friendly alternatives while reducing reliance on traditional energy sources and mineral resources, thereby providing practical solutions to environmental and energy challenges. Overall, this review not only provides a comprehensive comparative analysis of different LBC preparation methods, but also facilitates a deeper understanding of the advantages and limitations of these methodologies when it comes to developing high-value materials for sustainable applications.

## 1. Introduction

With the burgeoning global population and escalating urbanization, energy demand has undergone a relentless surge, thereby straining the availability of conventional energy sources [1,2,3]. Predominantly, fossil fuels have been the cornerstone of the global energy landscape. However, their consumption significantly contributes to carbon dioxide emissions, exacerbating climate change and environmental degradation [4]. Moreover, the finitude of fossil fuel reserves coupled with escalating prices underscores the urgent need for alternatives. In this milieu, heightened emphasis on environmental stewardship has catalyzed the exploration of sustainable, renewable energy sources. Renewable energy and sustainable technologies offer a promising avenue for mitigating environmental impacts while reducing dependence on fossil fuels. In this context, biomass, as a typical sustainable biological resource, refers to various organisms, including animals, plants, and microorganisms [5,6], that facilitate the conversion of carbon dioxide, water, and sunlight through photosynthesis [7]. In stark contrast to the finite and environmentally taxing fossil fuels, biomass is widely available in nature and harbors immense potential for energy production, as well as the synthesis of chemicals and other products [8,9,10]. The utilization of this renewable resource to produce carbon materials, such as biochar [11,12] and carbon nanotubes [13,14], as well as low-emission fuels [15], plays an increasingly important role in gradually replacing traditional fossil fuel processes and has garnered significant attention in the academic community. Among these, biochar has important value and potential in environmental governance, increasing agricultural yield, sustainable energy, industrial applications, and waste management due to its unique physical and chemical properties.

**Figure 1:**
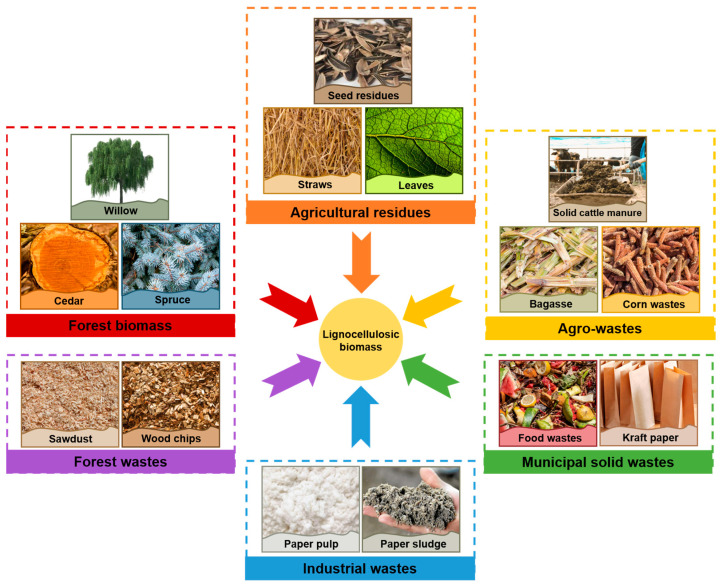
The main sources of lignocellulosic biomass.

The prevailing overdependence on fossil fuel resources can be significantly alleviated through the strategic conversion of lignocellulosic biomass (LB). This type of biomass, characterized by its composition of plant cell walls rich in lignocellulosic fibers [16], can be sourced from a variety of materials (Figure 1), including agricultural residues [17], agro-wastes [18], forest biomass [19], forest wastes [20], municipal solid wastes [21], and industrial wastes [22]. At its core, LB comprises three primary components: lignin, cellulose, and hemicellulose, with lignin assuming a pivotal role. This central component reinforces the cell structure of plant cell walls, conferring attributes of hardness, durability, and resistance to biodegradation. Lignin is categorized into three major chemical classes: guaiacyl lignin, syringyl lignin, and p-hydroxyphenyl lignin. The polymerization of these classes forms a complex, three-dimensional network that enhances the strength and stability of the cell wall. Notably, compared to other biomass components, lignin’s high carbon content renders it an ideal precursor for LB-derived biochar (LBC) production [23,24].

LBC represents a specific category of carbon-based materials derived from LB. The properties of the obtained LBC are significantly influenced by several factors, such as the selection of the carbonaceous precursor, the methods employed for carbonization and activation, and various synthesis conditions. These factors collectively shape the physical and chemical characteristics of LBC. Due to its unique porous structure, expansive surface area, and substantial energy content, LBC finds wide-ranging applications in the fields of agriculture, environmental science, and energy production. This environmentally friendly alternative exhibits immense potential in replacing traditional fossil fuel resources, thereby aligning with the principles of sustainability and environmental preservation. By harnessing LBC, we can promote a transition towards cleaner and more sustainable energy sources while simultaneously addressing environmental concerns.

This review comprehensively summarizes the conversion technologies of LBCs in recent years, mainly focusing on their carbonization and activation. Furthermore, it summarizes the wide applications of LBCs in agriculture, environment, energy, and other fields, demonstrating that they are promising in addressing current and future environmental and energy challenges. Overall, it can offer an extensive comparative analysis of the advantages and disadvantages of different preparation methods of LBCs, thus contributing to a deeper understanding of their strengths and limitations in sustainable utilization.

## 2. Preparation of Lignocellulosic Biomass-Derived Biochar

### 2.1. Carbonization Methods

LB, especially waste or low-value types, typically undergoes carbonization during experimental and industrial stages. This treatment involves physicochemical transformations via heating, inducing pyrolysis and structural rearrangement. Some elements are converted into volatile gases and released, transforming the original LB into LBC, a carbonaceous product. To further enhance the performance and value of LBC, activation or modification treatments are often applied, increasing its pore structure and surface functional groups to improve its adsorption capacity and reactivity. This method can produce biomass-based activated carbon with a high specific surface area and mechanical strength [25,26].

#### 2.1.1. Direct Carbonization

The broad concept of carbonization includes calcination and pyrolysis, differentiated by the presence or absence of oxygen. Calcination involves reactions with oxygen, while pyrolysis occurs without it. Pyrolysis is the most common and simplest carbonization method for LB due to its operational simplicity, cost-effectiveness, and ability to produce materials with a larger specific surface area and well-developed pore structures. However, it has drawbacks, such as forming biochars with non-uniform morphology and higher impurity content, which needs ongoing optimization in pyrolysis research.

**Figure 2:**
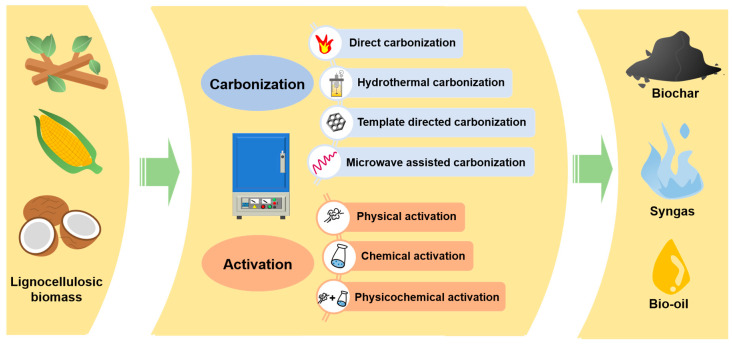
The preparation of lignocellulosic biomass-derived biochar.

Temperature is crucial in determining the formation of primary products and by-products in pyrolysis [27]. Different biomass sources and pyrolysis methods can lead to the production of desired biochar, bio-oil, or unintended volatile gases (Figure 2). High temperatures can cause excessive biomass decomposition, reducing the as-prepared biochar adsorption performance. Conversely, low temperatures can produce biochar with smaller specific surface areas and underdeveloped pore structures. Therefore, an appropriate pyrolysis temperature needs to be selected for the structures and applications of LBC. For example, Bong et al. [28] explored optimal pyrolysis conditions for banana peels, including the selection of the operating temperature, residence time, and heating rate, demonstrating the significant impact of pyrolysis temperature on biochar yield and stability. Sahoo et al. [29] investigated the pyrolysis process of bamboo and pigeon pea stalk at different temperatures (400–600 °C), showing that the yield of produced biochar decreased at 600 °C. However, the biochar still had low volatile content, high fixed carbon content (81.85–85.68%), and high ash recovery rates with extensive agricultural applications. Additionally, the study concluded that bamboo exhibited a higher biochar yield, possibly due to its higher content of lignocellulosic fibers and cellulose. Hong et al. [30] researched straw pyrolysis by investigating product composition, energy conversion, and structural characteristics. The research findings indicated that increasing the pyrolysis temperature reduced the biochar yield and energy conversion efficiency while the fixed carbon content increased. Additionally, higher temperatures increased the specific surface area of biochar, decreased pore size, and weakened the intensity of functional groups. Furthermore, Qin et al. [31] found that the biochar yield negatively correlated with increasing residence time.

Furthermore, studying the pyrolysis rates is essential. Current works mainly focus on slow pyrolysis and fast pyrolysis methods for LB. Slow pyrolysis is characterized by slow heating rates (0.02 °C/s to 1 °C/s), long residence time (several hours to days), and low temperature (300–700 °C). It is known that the yields of produced LBC characterized by a high lignin content will be higher compared to other biomass carbon materials. Therefore, slow pyrolysis is often employed in the preparation of LBC. For example, Adekanye et al. [32] obtained biochar from corn cob waste through slow pyrolysis, finding that the biochar yield decreased as the temperature increased. The heating rate significantly influences the physicochemical properties of biochar, with a maximum specific surface area of 281.8 m^2^/g achieved when the temperature was slowly raised to 500 °C. Farobie et al. [33] conducted slow pyrolysis on seaweed in a batch reactor, noting a decrease in the H/C and O/C atomic ratios of biochar, which indicated dehydration and decarboxylation reactions of LB. Additionally, the experiments showed that the heating value of the biochar (23.12–25.89 MJ/kg) increased with temperature, suggesting its potential as a solid fuel compared to low-rank coal. However, slow pyrolysis has limitations in long processing cycles and low energy efficiency, leading researchers to employ auxiliary techniques like vacuum pyrolysis, catalytic pyrolysis, and microwave pyrolysis to overcome these drawbacks.

Fast pyrolysis, characterized by rapid heating rates (>2 °C/s), short residence time (<10 s), and a wide temperature range (300–1000 °C) [34,35], efficiently produces bio-oils but reduces the biochar yield. Therefore, the feedstock types, reaction environments, and operating conditions are crucial in the production and composition of LBC during oxygen-free pyrolysis.

#### 2.1.2. Hydrothermal Carbonization

**Figure 3:**
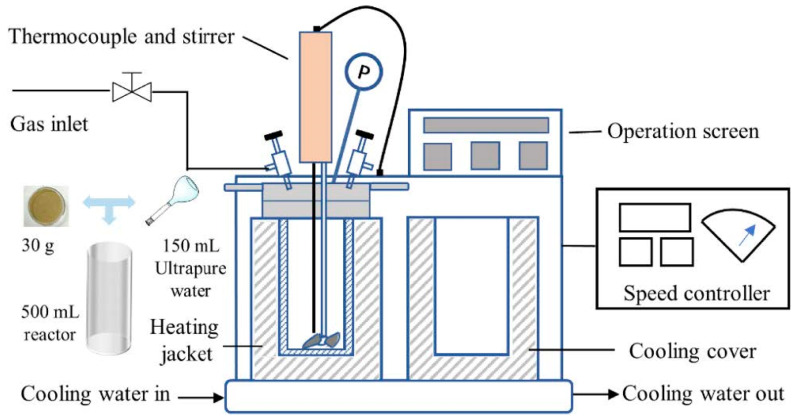
Schematic diagram of the lab-scale hydrothermal carbonization reactor (P: pressure gage). Reproduced with permission [39], Copyright 2022, Elsevier.

Hydrothermal carbonization (HTC) is a process that involves the mild hydrothermal reaction of biomass mixed with water in a specific ratio under certain pressure conditions (as shown in Figure 3). While water acts as an efficient heat transfer medium, mass transfer limitations can occur due to significant variations in particle size or if the reaction time is too short. Additionally, HTC’s products may contain potentially harmful compounds like metals and polycyclic aromatic hydrocarbons, which could limit the use of the products as soil amendments [36]. The HTC process typically includes hydrolysis, dehydration, decarboxylation, condensation, and aromatization reactions. The as-produced biochar, often referred to as hydrochar, tends to exhibit better physical and chemical properties than the biochar obtained through direct pyrolysis, making it an alternative to the traditional slow pyrolysis of LB. For instance, Regmi et al. [37] prepared hydrothermal activated carbons from switchgrass using the HTC method, which exhibited excellent adsorption properties. The rapid pyrolysis process led to the formation of abundant pores and functional groups on the surface of the activated carbon, resulting in a specific surface area of 726 m^2^/g, which enabled the effective adsorption of copper and cadmium from aqueous solutions. This work shows that hydrochar can retain oxygen and nitrogen elements from the feedstock, resulting in its surfaces having rich functional groups [38], which enhances its adsorption capacity.

Furthermore, many researchers have explored experiments involving HTC of LB in recent years. For example, utilizing bamboo pulp fibers as a raw material [40], studies on HTC with the aid of hydrochloric acid (HA) and phosphoric acid (PA) indicated that higher concentrations of HA could lead to a greater yield of regular, spherical porous carbon microspheres compared to PA. Researchers conducted HTC and direct pyrolysis on hornbeam wood chips from 225 °C to 575 °C and analyzed the produced biochar and hydrochar [41]. The results revealed that biochar obtained through pyrolysis had an incrementally higher fixed carbon content with rising temperatures. The highest calorific value for pyrolysis-produced biochar was noted at 575 °C, reaching 32.51 MJ/kg. Similarly, experiments with sawdust of deciduous trees under both direct carbonization and HTC showed that both the yield and calorific value of the biochar were enhanced when a consistent residence time of 1 h was maintained, and the operational temperatures were improved from 190 °C to 290 °C [42]. Notably, HTC was effective in biomass conversion even at lower temperatures, a result not replicated by direct carbonization.

HTC offers several advantages over traditional pyrolysis methods. A key benefit is its ability to process wet feedstock without pre-drying, making it more efficient in handling moist materials. Additionally, HTC can reduce the content of elements like alkali, alkaline earth metals, and heavy metals, thereby enhancing its calorific value [43]. Despite these benefits, HTC is still an emerging technology and faces challenges such as lower selectivity and the potential generation of unwanted by-products [44]; thus, further research is still needed. In recent years, researchers have focused on reducing the reaction temperatures for LB, targeting around 200 °C for materials like straw and poplar leaves [24]. This development is significant as it addresses the high-temperature and energy consumption limitations of traditional HTC methods. By achieving low-temperature HTC, the process can effectively bypass restrictions imposed by the crystalline structure of cellulose in high-cellulose-content biomass. This method facilitates dehydration carbonization, the formation of unsaturated bonds, and the aromatization of cellulose and hemicellulose, paving the way for creating carbon materials from LB with a potential carbon-negative impact.

#### 2.1.3. Template-Directed Carbonization

Template-directed carbonization (TDC) involves using a substrate material as a template to synthesize carbon materials on its surface. These substrate materials can be silica dioxide, micelles, and metal complexes, with adjustable pore sizes according to experimental requirements. Hollow carbon materials are obtained by etching the template or applying high-temperature heating. Compared to pyrolysis and HTC, TDC processes are more complex, requiring longer reaction times (1–30 h) and intermediate stages. However, TDC methods offer significant advantages: the produced LBC or activated carbon has more controllable pore sizes and stable structures [45]. Additionally, the templates used are relatively inexpensive and readily available, and they can avoid any activation or functionalization processes of the biomass, thereby reducing production costs. While still in the development stage, TDC methods have shown potential applications in various fields, such as bio-derived catalysts for biodiesel synthesis and nanostructured porous carbons for high-performance supercapacitor electrode materials.

**Figure 4:**
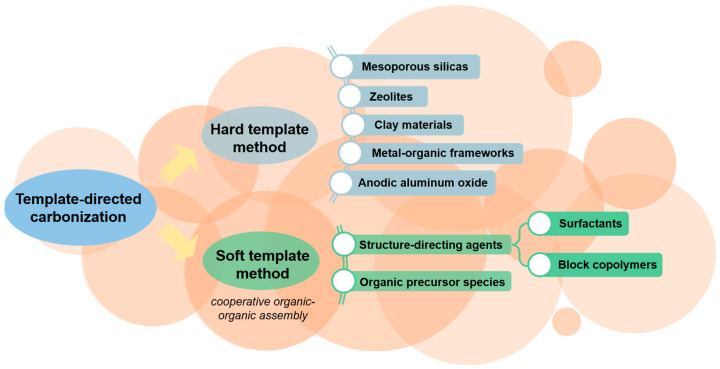
Template-directed carbonization of products derived from lignocellulosic biomass.

TDC methods are categorized into hard template synthesis and soft template synthesis (Figure 4) [46]. Hard template synthesis prepares uniform and ordered porous carbon materials but involves complex processes and toxic chemicals, limiting large-scale production. Soft template synthesis, on the other hand, uses the cooperative self-assembly of hydrophobic and hydrophilic molecules (like surfactants and block copolymers) with carbon precursors (such as phenolic resins). It is more suitable for creating ordered mesoporous carbon materials. In the soft template method, molecules or components in the template interact through hydrophobic, hydrophilic, and electrostatic interactions, and hydrogen bonding. Under suitable conditions, ordered micelles can spontaneously form templates in the aqueous solvent and combine with the carbon precursor to form heterogeneous composites. During carbonization, porous carbon materials with specific mesoporous structures are formed. Soft template methods are more straightforward than hard template synthesis as they do not require corrosive acids and bases to remove the template. However, challenges exist, such as most LB waste being insoluble and unable to form stable colloidal dependence with soft templates. Additionally, the surfactants used are expensive and non-recoverable, limiting the large-scale application of the soft template method [47]. Therefore, addressing these issues is crucial for applying the soft template method in preparing porous carbon materials.

**Figure 5:**
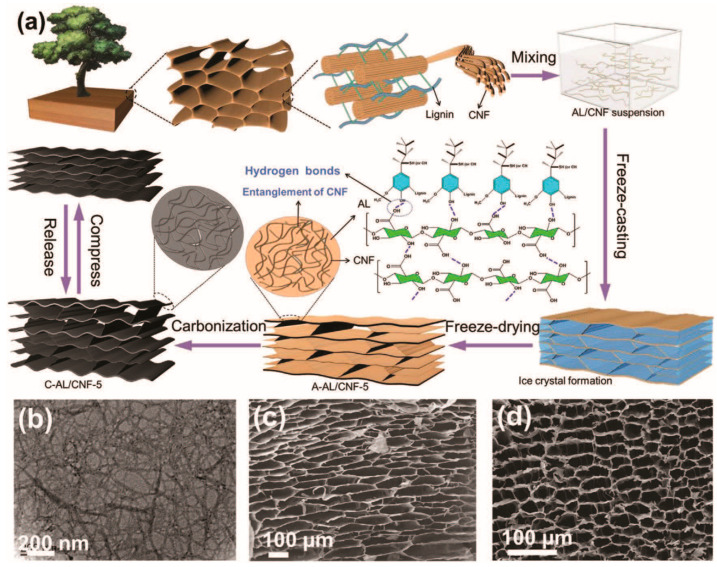
(**a**) Illustration of fabricating wood-derived carbon aerogel C-AL/CNF-5. (**b**) Transmission electron microscope image of CNF. SEM images of (**c**) A-AL/CNF-5 and (**d**) C-AL/CNF-5. Reproduced with permission [49], Copyright 2020, WILEY-VCH.

Liu et al. [48] employed a dual-template-directed carbonization method, where an Al(III)-based metal-organic framework was used as the free-standing template (hard template). Additionally, Pluronic F127 was used as the microstructure-directing agent (soft template), using banana peel to synthesize biomass-derived catalysts. The hard template can create macropores, while the soft template can generate mesopores [46]. The pores produced by the hard template had larger volumes but were relatively unstable, whereas the soft template provided a more stable mesoporous structure with lower overall pore volume and surface area. Leveraging the advantages of both hard and soft templates, the dual-template approach balances pore structure and stability, which is crucial for specific applications. However, further research is needed to validate the feasibility and effectiveness of this method under different conditions and in various application domains. Moreover, novel templates like ice templating have recently been employed to fabricate functional nanoporous carbons. As illustrated in Figure 5, Chen et al. [49] combined cellulose nanofibers (CNFs) with alkali lignin (AL) from wood to form an aerogel (A-AL/CNF-5) through freeze drying and prepared its carbon aerogel (C-AL/CNF-5) through carbonization. Ice crystals directionally grow to extrude the highly intertwined CNFs during directional freeze casting, wherein an ordered tracheid-like stable structure, high carbon content, a specific surface area of 950.4 m^2^/g, and a wide range of pores were obtained. This innovative and straightforward strategy effectively tackles some of the challenges associated with traditional TDC, offering researchers a fresh perspective and a new approach to consider in the field of TDC.

#### 2.1.4. Microwave-Assisted Carbonization

**Figure 6:**
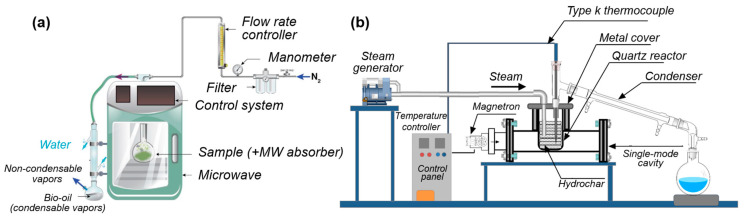
(**a**) Experimental set-up for microwave-assisted pyrolysis of pine wood sawdust biomass. Reproduced with permission [56], Copyright 2020, MDPI. (**b**) Schematic diagram of single-mode microwave hydrothermal carbonizer. Reproduced with permission [57], Copyright 2022, Elsevier.

Microwave-assisted carbonization (MAC) is a novel technique for carbonizing LB, bearing similarities to pyrolysis but differing primarily in the heating mechanism. Unlike traditional pyrolysis, which relies on direct heating, MAC utilizes microwave heating. Microwaves, a form of non-ionizing radiation comprising two perpendicular electromagnetic wave components, efficiently convert electromagnetic energy into heat, thus facilitating the heating of LB. Recognized as an energy conversion process distinct from conventional heat transfer through a medium [50], MAC offers several advantages over traditional pyrolysis, including internal heating, reduced energy consumption, and shorter processing times [51]. Standard MACs include direct microwave-assisted pyrolysis and microwave-assisted hydrothermal carbonization (Figure 6). The microwave system enables more uniform heating of LB, leading to LBC with more consistent chemical properties. Moreover, MAC excels in controllability and energy efficiency, as the rapid heat generation by microwaves minimizes environmental energy loss [52]. MAC has been successfully applied in the production of biochar and biofuels. Many researchers have conducted extensive research on MAC, covering aspects such as its principles, mechanisms, and pyrolysis conditions, the influence of different variables on product distributions, and comparisons with traditional pyrolysis methods [53,54,55,56].

For instance, Nair et al. [58] demonstrated the effectiveness of MAC in producing high-specific-surface-area biochar from *Prosopis juliflora* biomass, achieving a surface area of 357 m^2^/g at 600 W microwave power. Luo et al. [59] used biochar obtained through MAC as a catalyst for tar removal, finding that the biochar surface had a higher concentration of oxygen functional groups and alkali metal elements, which enhanced its effectiveness. Paramasivan et al. [60] focused on the use of microwave-assisted pyrolysis and its reactor, discussing its advantages over traditional methods, particularly in improving the quality of biofuels and its potential role in sustainable agriculture as a soil amendment. Yek et al. [57] developed a single-mode microwave hydrothermal reactor capable of producing hydrochar at various temperatures (150–300 °C) within just 10 min under steam sweeping conditions (Figure 6b). The resulting hydrochar showed a higher yield and lower moisture, volatile matter, and ash content, thereby proving beneficial for the dechlorination of domestic water. Hessien et al. [61] introduced a facile method to produce hydrochar from pomegranate peel waste using the microwave-assisted hydrothermal carbonization method at 200 °C for 1 h with a 1:10 mass ratio of peel to water. This method synthesized amorphous, oxygen-rich, porous hydrochar efficiently and in an eco-friendly way. Hidalgo et al. [62] successfully synthesized carbon nanotubes from agricultural and industrial waste materials like wheat straw and hazelnut shells using MAC at temperatures of 400 °C and 600 °C, finding that the pyrolysis temperature significantly influenced the physicochemical properties of the carbon nanotubes. The experiments conducted at 600 °C resulted in higher concentrations of carbon nanotubes in the produced biochar. Remarkably, carbon nanotubes synthesized from hazelnut shells and wheat straw exhibited a higher degree of graphitization, demonstrating excellent quality characteristics.

Moreover, Zhang et al. [63] prepared hydrochar from various corn residues, showing that the heating value of hydrochar significantly increased to about 20.7 MJ/kg under microwave-assisted hydrothermal conditions at 230 °C, which marked a substantial improvement compared to the raw materials. Furthermore, the apparent activation energy of the hydrochar increased, which can be attributed to the increased crystallinity and the higher number of C=C and C=O bonds during the microwave-assisted hydrothermal process. The study also demonstrated that deoxygenation, dehydration, and decarboxylation contributed to carbon enrichment during microwave-assisted hydrothermal processes. These findings provide the necessary experimental and theoretical foundations for the preparation of high-quality hydrochar using MAC techniques.

Despite its benefits, MAC faces challenges, including the need for microwave absorbers in the feedstock and hotspot phenomena [64]. Currently, the technology can be categorized into non-catalytic and catalytic-assisted microwave pyrolysis, with the former capable of producing biochar at high yields and low power. Factors such as power and microwave time significantly affect the yield of LB, like corn cobs, in non-catalytic microwave pyrolysis. Lower power can accelerate the carbonization process in a short time, while higher power can promote syngas production [65]. The performance of catalytic-assisted microwave pyrolysis has also been widely discussed, emphasizing the importance of different catalysts in improving energy efficiency and selective distribution [66,67]. Typical catalysts include soluble inorganic substances, metal oxides, microporous materials, and carbonaceous materials [68,69]. From an economic perspective, the cost of MAC technology is generally higher than that of traditional methods, but, with decreasing biomass feedstock costs and improvements in microwave equipment, the expense is expected to be reduced. However, the challenges remain in scaling up MAC for commercial applications, including equipment costs, energy efficiency, and feasibility. Therefore, further research and engineering practices are essential to advancing the development and application of MAC technologies. Table 1 shows a comparison of different carbonization methods of LBC. Different biochar synthesis technologies can significantly decrease the overall quantity of LB waste requiring ultimate disposal and allow for more effective and controlled management, ensuring compliance with pollution control regulations.

### 2.2. Activation Methods

During the carbonization process of LB, the resulting products often lack a substantial pore structure. An activation treatment can be performed on the carbonized product to enhance the porosity and specific surface area and introduce functional groups on its surface that can enhance its performance [70]. Activation refers to the process of creating pores in the carbonaceous materials. The crucial aspect is determining the appropriate temperature and duration to produce activated carbon with a significant surface area, sufficient pore formation, and surface functional groups while also maintaining its mechanical stability. Standard activation methods include physical, chemical, and physicochemical activation [47,71,72,73,74,75].

#### 2.2.1. Physical Activation

Physical activation, also known as gas activation, employs oxidizing gases like water vapor, carbon dioxide, oxygen, air, or their mixtures as activating agents to partially oxidize LBC at temperatures ranging from 600 °C to 1200 °C, thereby forming a porous structure. The essence of physical activation is the process of etching and pore formation on the carbon framework by these oxidizing gases [76]. For example, Sumathi et al. [77] used carbon dioxide as an activating agent to activate optimally treated palm shell biochar, achieving a specific surface area of 973 m^2^/g, a total pore volume of 0.78 cm^3^/g, and a micropore fraction of 70.5%. The palm-shell-activated carbon with a high specific surface area and micropore fraction exhibited excellent SO_2_ adsorption performance. Similarly, Zhao et al. [78] prepared activated carbon with a high specific surface area and excellent adsorption performance using walnut shells and CO_2_ at 900 °C. The activated carbon reached 1228 m^2^/g and the maximum water vapor adsorption capacity of 0.3824 g/g. Sakanishi et al. [79] conducted pyrolysis and activation of red pine wood using CO_2_ at 800 °C, resulting in activated carbon with the adsorption capacity volume of 0.255 cm^3^/g for H_2_S. These investigations showed that introducing these oxygen-rich gases during the high-temperature treatment of LB raw materials helps generate carbonaceous materials with micropores and mesopores, which contributes to the adsorption of various gases.

**Figure 7:**
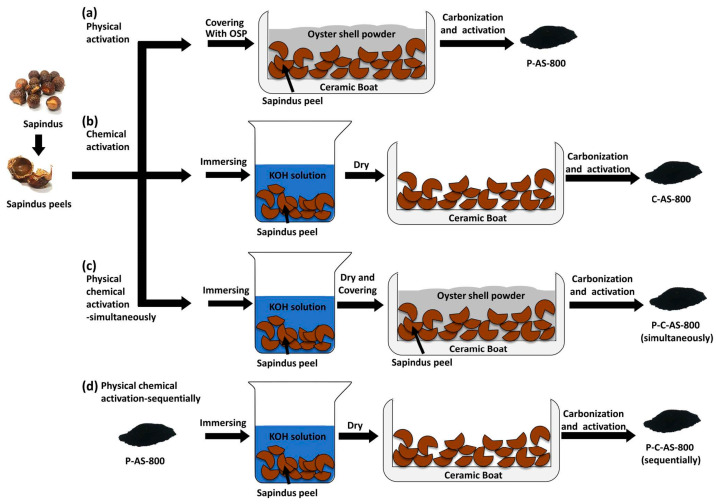
Schematic diagram of the carbonized Sapindus sample preparations: (**a**) physical activation, (**b**) chemical activation, (**c**) simultaneous physical and chemical activation, (**d**) sequential physical and chemical activation. Reproduced with permission [72], Copyright 2023, Elsevier.

In addition, researchers have recently explored the use of non-traditional physical activating agents for the activation of LBC. Hsiao et al. [72] innovatively used soapberry pericarp as the carbon source due to its high content of N and O carbides and employed oyster shell powder as the activating agent (Figure 7). The cost of the physical activation process was reduced by using the CO_2_ gas generated from the thermal decomposition of oyster shell powder instead of water vapor, air, and other traditional gases. Physical activation has advantages like simplicity and no secondary pollution, but it also has drawbacks such as long activation time, high energy consumption, and low activated carbon yield [80].

#### 2.2.2. Chemical Activation

Chemical activation is a prevalent method for preparing activated carbon. It involves mixing an activating agent with the raw material in a specified ratio, ensuring complete impregnation, followed by carbonization and activation treatments under inert gas protection. The key to this process is the penetration of the activating agent into the internal structure of the carbon particles and its interaction with internal impurities, such as carbon, hydrogen, and oxygen, resulting in the formation of activated carbon with abundant pore structures and well-developed porosity. Compared with the physical activation methods, chemical activation methods are widely used in the preparation process due to some advantages, such as higher activation efficiency, less carbon burn-off, higher yield of activated carbon, relatively shorter operating time, lower operating temperature, and higher pore volume [81]. The widely used chemical activating agents are acids or alkali metal hydroxide/salt solutions, including phosphoric acid (H_3_PO_4_), zinc chloride (ZnCl_2_), potassium hydroxide (KOH), sodium hydroxide (NaOH), sulfuric acid (H_2_SO_4_), and ferric chloride (FeCl_3_) [75]. Different activating agents play different roles in the activation process, resulting in significant differences in their activation mechanisms. Therefore, selecting the appropriate activating agent for preparing high-performance activated carbon is crucial.

For example, Tsai et al. [82] prepared high-surface-area activated carbon from cocoa bean shells using KOH as the activating agent. At 800 °C, the resulting activated carbon exhibited a specific surface area of 1800 m^2^/g and a total pore volume of 0.95 cm^3^/g, proving effective for removing organic pollutants from water. Davarnejad et al. [83] employed NaOH as the activating agent to prepare activated carbon from grape stalk powder under activation at 550 °C for 120~270 min, resulting in a material with abundant surface pores, large specific surface area, and functional groups, ideal for adsorbing methylene blue dye. Taheripak et al. [84] prepared activated carbon derived from oak seed shells using phosphoric acid at 450 °C for the adsorption and removal of crude oil from wastewater. Chen et al. [85] selected biochar derived from peanut shells and used ZnCl_2_ as the activating agent to prepare active carbon-sulfur composite materials for rechargeable lithium-sulfur batteries. This activated carbon exhibited a rich microporous structure on its surface, making it more suitable for reactions in lithium-sulfur batteries. In these methods of activating biochar with a single chemical activator, the researchers chose different activators, resulting in different properties and applications of the biochar prepared.

**Figure 8:**
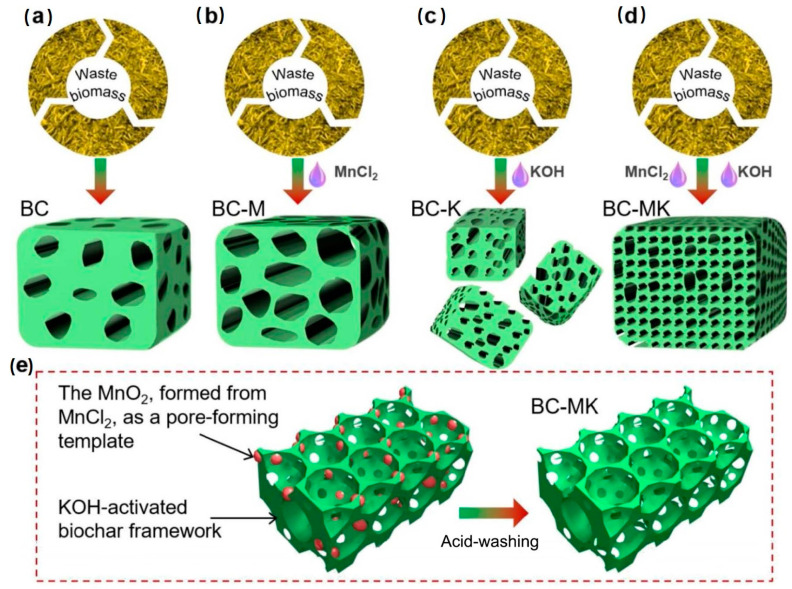
Schematic illustration of the preparation of banana-peel-derived biochar and its activated counterparts by different chemical activation treatments. (**a**–**d**) Schematic presentation of porous structure evolution from the BC (**a**), BC-M (**b**), BC-K (**c**), to BC-MK (**d**) samples. (**e**) Schematic models showing the templating role of MnCl_2_ in pore formation within the biochar framework. Reproduced with permission [86], Copyright 2023, Elsevier.

Moreover, with the continuous improvement in chemical activation methods, the dual-chemical strategy has become more popular. Due to the lack of efficient and direct conversion processes, Zhang et al. [86] utilized dual-chemical (MnCl_2_/KOH) activation to directly convert banana peels into high-porosity biochar, achieving a specific surface area of 1276.63 m^2^/g, which was superior to single-chemical activation. As shown in Figure 8, the biochar (BC) activated by MnCl_2_, KOH, and MnCl_2_/KOH was noted as BC-M, BC-K, and BC-MK, respectively. This work showed that KOH exhibited a strong etching effect on the biochar framework, simultaneously generating gases that facilitated the formation of primary porous structures. The presence of MnCl_2_ was highly effective in revealing the internal components of the biomass and facilitating a uniform chemical reaction with KOH. Furthermore, during high-temperature pyrolysis in the presence of KOH, MnCl_2_ undergoes conversion into MnO_2_ species within the biochar matrix, allowing it to serve as a template. Subsequent pickling processes removed these substances, further enhancing the porous structure within the inner layers of the biochar matrix. Moreover, the biochar produced with adjusted pore volume and specific surface area was demonstrated to have effective adsorption of tetracycline antibiotic contaminants.

Physical activation is simpler and cleaner, and there is no need for chemicals or wastewater treatment. Chemical activation is more effective for specific applications requiring unique pore structures. However, it involves challenges like chemical consumption, equipment corrosion, chemical recovery, and secondary pollution, increasing wastewater treatment costs and limiting industrial-scale applications [87]. Therefore, the choice of activation method should consider process requirements, cost-effectiveness, and environmental impacts.

#### 2.2.3. Physicochemical Activation

Physicochemical activation is a hybrid approach that synergizes the strengths of both physical and chemical activation techniques. In this process, the impregnation of the activating agent allows for a uniform distribution within the raw material, increasing the contact area between the activating agent and the raw material. Subsequent heating promotes the activation of the activating agent, inducing chemical reactions with the raw material, thereby enhancing the pore structure and surface activity of the activated carbon. Additionally, introducing gas at high temperatures during the physical activation step further enlarges the pore size and improves pore distribution, augmenting the adsorption capabilities of the activated carbon. Table 2 shows a comparison of different activation methods of LBC.

**Figure 9:**
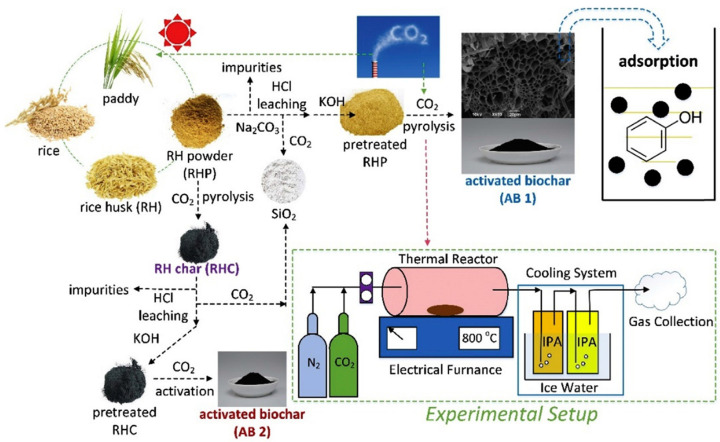
Schematic of activated biochar preparation from rice husk via CO_2_ pyrolysis. Reproduced with permission [89], Copyright 2018, Elsevier.

In the research conducted by Patel et al. [88], an economically efficient method for synthesizing activated porous carbon using pine sawdust as the raw material was reported, and a hybrid synthesis approach was proposed by involving physicochemical activation with KOH + CO_2_. This dual-agent activation effectively transformed pine sawdust into an activated porous carbon material with a high surface area. The researchers also compared its CO_2_ adsorption capture capacity with activated carbon obtained solely through chemical or physical activation. Shen et al. [89] carried out a comparative study on the effects of one-step and two-step methods for preparing activated biochar from rice husk via KOH-catalyzed pyrolysis in a CO_2_ environment (Figure 9). The one-step method involved direct pyrolysis of rice husk with KOH under CO_2_, forming activated biochar. This method simplified the preparation process and achieved higher yields. Conversely, the two-step method involved initial pyrolysis of rice husk followed by physicochemical activation with KOH to produce activated biochar. Krishnamoorthy et al. [90] utilized date pits as the raw material and prepared activated carbon through the activation method using phosphoric acid and nitrogen gas. This method offered the advantages of low-cost and abundant raw materials while producing activated carbon materials with excellent adsorption performance. Additionally, the prepared activated carbon exhibited high efficiency in adsorbing Pb^2+^ in aqueous solutions, demonstrating its effectiveness in removing Pb^2+^ from wastewater.

## 3. Applications of Lignocellulosic Biomass-Derived Biochar

**Figure 10:**
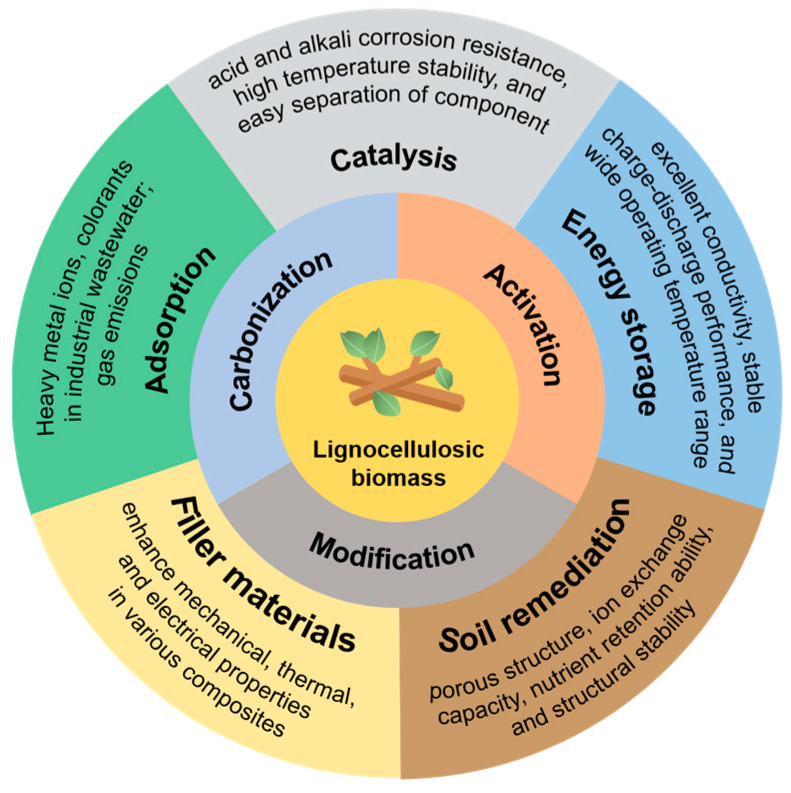
Applications of biochar derived from lignocellulosic biomass.

Utilizing LB to produce carbon materials is a viable choice due to the high carbon content present in lignin. Various sources of LB, such as durian shells [91,92], coconut shells [93,94,95], and food waste [96], have been effectively used to prepare activated carbon with high adsorption properties, based on different advantages of carbonization and activation methods. LBC finds wide applications in the industrial and residential sectors (Figure 10). In wastewater treatment [74,97], it is crucial to remove pollutants from water. It can also be employed in air pollution control to purify harmful gases and particulates from the air [98]. In the petroleum refining industry, this activated carbon is used to adsorb and separate impurities from petroleum products [99], enhancing the quality and purity of the final products. Additionally, it is significantly applied in energy storage [47], such as in electrode materials for supercapacitors and lithium-ion batteries. Furthermore, it can be utilized in catalytic processes as catalysts or catalyst-support materials in chemical reactions [74]. In summary, LBC is highly versatile, finding uses in wastewater treatment, air pollution control, petroleum refining, energy storage, and catalysis. Its application not only offers sustainable and eco-friendly alternatives, but also helps reduce reliance on traditional fossil fuels and mineral resources, thereby providing practical solutions to environmental and energy challenges.

### 3.1. Adsorption

Industrial wastewater often contains heavy metal ions such as Hg^2+^, Pd^2+^, and Cr^6+^, which can negatively impact human health even at low concentrations, leading to cancer, respiratory diseases, and cardiovascular diseases. Moreover, the discharge of heavy metals into water bodies poses a threat to aquatic organisms. When treating inorganic industrial wastewater, LBC is commonly used to adsorb and remove heavy metal ions from water [100]. The adsorption capacity of LBC is influenced by the biomass feedstock, conversion techniques, and processing conditions. Owing to its high specific surface area, porous structure, and surface oxygen functional groups, LBC is particularly efficient in removing heavy metals from wastewater. Liu et al. [101] demonstrated that LB prepared from corn cobs exhibited a maximum adsorption rate of 97.2% for mercury ions. Zhao et al. [102] explored the adsorption performance of different LBCs derived from poplar, corn, and *Brassica napus* for multiple heavy metal ions (Pb^2+^, Cr^3+^, Cu^2+^, and Cd^2+^), highlighting the importance of the complexation, porous structure, and cation exchange in the process. Additionally, Zamani et al. [103] reported using biochar derived from oil palm empty fruit bunches to remove zinc ions, achieving a biochar yield of 25.5 wt% and an adsorption capacity of 15.2 mg/g for Zn^2+^.

Apart from heavy metals, LBC is also used to adsorb other pollutants in industrial wastewater, such as colorants, odors, inorganic compounds, and organic substances. For instance, LBC effectively treats dyeing and printing wastewater, removing stubborn dyes like methylene blue [104]. Wang et al. [105] utilized waste-bamboo-derived biochar as an adsorbent for methylene blue dye, achieving a maximum capacity of 1100 mg/g due to its graphene-like structure with abundant micropores and high specific surface area. Furthermore, LB has also shown high adsorption efficiency for pharmaceutical waste. Essandoh et al. [106] successfully removed pharmaceutical compounds such as salicylic acid and ibuprofen from aqueous solutions using pine wood biochar, with adsorption capacities of 22.7 mg/g and 10.7 mg/g, respectively.

In the realm of industrial gas emissions, LBC also shows promising adsorption capabilities. Its microporous structure and surface functional groups make it suitable for removing odorous gases and recovering exhaust gases generated by factories. Rashidi et al. [107] prepared high-carbon content and thermally stable activated carbon from palm kernel shells to absorb CO_2_ gas produced in industries, achieving an adsorption capacity of 2.13 mmol/g. Similarly, Mukherjee [108] showed that the biochar derived from waste coffee grounds had enhanced structural and physicochemical properties for excellent CO_2_ capture performance. Igalavithana [109] compared the CO_2_ adsorption performance of pine sawdust and paper mill sludge biochar at 25 °C, with the former showing superior adsorption performance (0.67 mmol/g) due to a higher specific surface area and better-developed microporous structure. These research findings indicate that LBC holds potential for application in industrial gas emissions and can be utilized as an adsorbent for removing gaseous pollutants.

In summary, LBC can offer advantages as an adsorbent, including ease of operation, low raw material cost, and wide availability of resources. Its hydrophilic functional groups and porous structure enable it to efficiently adsorb various impurities, including heavy metal ions, organic solvents, dyes, and CO_2,_ in both wastewater and industrial exhaust gases.

### 3.2. Catalysis

LBC not only possesses excellent carrier properties, but also exhibits favorable physicochemical properties such as acid and alkali corrosion resistance, high temperature stability, and easy separation of components. Additionally, its richness in functional groups further makes it widely applicable in the realm of catalyst supports [74]. The catalytic efficiency of LBC is greatly influenced by its porosity and specific surface area, which are crucial for enhancing mass transfer and catalytic selectivity [110]. The effectiveness of LBC catalysts can vary significantly based on the source of LB and the specific conditions under which it is activated, as each type of LB has distinct physicochemical characteristics [111].

For example, Saeed et al. [112] prepared catalytic biochar from coconut shells through pyrolysis at 500 °C and used it to promote enzyme production in solid-state fermentation. The study showed that the highest yield of *β*-glucosidase, reaching 92 IU/gds, was achieved using a biochar catalyst concentration of 2.5 mg at 40 °C for 72 h. Jiang et al. [113] produced biochar-based catalysts having a high acid quantity from corn stalks, with specific surface areas ranging from 1120 m^2^/g to 1640 m^2^/g. These catalysts, used in the hydrothermal degradation of lignin, resulted in more aromatic compound production compared to non-catalyzed processes and were demonstrated to be reusable. Li et al. [114] compared the catalytic activities of LBCs derived from different biomasses, including corn stalk, reed, and water hyacinth, in tar removal, and found that adding biochar led to a tar conversion efficiency of 94.6%.

LBC offers a versatile and effective catalyst option due to its favorable physicochemical properties and richness of functional groups. The variation in catalytic performance based on the LBC type underscores the importance of selecting the right LBC for specific applications, particularly in processes like enzyme production, lignin degradation, and tar removal [115,116].

### 3.3. Energy Storage

Activated carbon derived from LB is highly valued in energy storage applications, mainly as electrode materials for supercapacitors. Its excellent conductivity, stable charge–discharge performance, and wide operating temperature range are complemented by a hierarchical structure that boosts the specific capacitance [117].

Zhang et al. [118] utilized reed residue waste to produce activated carbon for supercapacitor electrodes. Pyrolyzing at various temperatures, they found that carbon activated at 600 °C, with its pinecone-like porous nanostructure, high surface area (2074.72 m^2^/g), and large pore volume (0.93 cm^3^/g), showed outstanding electrochemical performance. Its electrochemical performance was evaluated through cyclic voltammetry, constant current charge–discharge, and electrochemical impedance spectroscopy. It exhibited a capacitance of 228 F/g at 1 A/g in a 6 M KOH electrolyte and maintained 98.1% capacitance retention after numerous cycles. Liao et al. [119] prepared nitrogen-doped biochar material by direct pyrolysis (550 °C) of reed stalks and melamine. The biochar material possessed microporous and mesoporous structures, as well as abundant active nitrogen functional groups, which facilitated the enhancement of ion transport and Faradaic capacitance. As an anode for supercapacitors in a 6 M KOH electrolyte, it exhibited a capacitance of 202.8 F/g at 1 A/g. Even at a higher current density of 20 A/g, it still maintained a capacitance of 158 F/g and 96.3% capacitance retention after 5000 cycles. Numee et al. [120] prepared activated carbon from durian rind using radiation treatment, simple HTC, and physical activation. They subjected the biomass powder to different doses of gamma radiation and electron beam irradiation, followed by HTC at 200 °C for 8 h, using a mixture of ZnCl_2_ and FeCl_3_ as the activating agent. Subsequently, the obtained hydrochar was pyrolyzed at 600 °C for 2 h under an argon atmosphere. This work found that irradiation significantly improved the Coulombic efficiency of the electrode material. The best-performing electrode material achieved a specific capacitance of 325.20 F/g at 1 A/g, with 94.79% retention after 10,000 cycles. Therefore, the results successfully demonstrated a promising method with high potential for large-scale production and application of LBC in energy storage technologies. This not only helps alleviate environmental issues but also contributes to the advancement of sustainable energy storage applications.

### 3.4. Other Applications

Beyond adsorption, catalysis, and energy storage, LBC can also be utilized as an eco-friendly filler material in various composites. It can enhance mechanical, thermal, and electrical properties when added to thermosetting materials, thermoplastics, and ceramic–polymer composites [121]. Due to its low density, LBC can be a sustainable alternative to inorganic fillers like glass or silica in polymer composites. Additionally, modifying its structure can significantly impact the filler properties in polymer composites. Cappello et al. [122] utilized biochar prepared from wood waste as a low-cost lubricating filler in polyester bio-composites. This biochar could be incorporated into the composites up to 20 wt.%, reducing the melt viscosity, acting as a lubricant, and enhancing the extrudability and injection molding performance of the composites at high temperatures.

Furthermore, LBC, such as olive tree prunings, can also be used in composite materials for effective electromagnetic shielding [123,124]. Additionally, due to its porous structure, ion exchange capacity, nutrient retention ability, and structural stability [125,126,127], LBC can be an ideal candidate for slow-release fertilizers in soil. By gradually releasing essential nutrients (such as nitrogen, phosphorus, and potassium) and organic carbon, LBC can enhance soil fertility, promote crop growth, and increase yields [128,129]. It can also be used as a fuel to produce energy and for coke in the industry field. Based on the publications mentioned above, it can be concluded that LBC is not only pivotal in environmental management, but also plays a significant role in advancing sustainable energy solutions and improving the properties of soil and various composite materials.

## 4. Conclusions

With the development of sustainable resources, the conversion technologies of LBC, including the carbonization and activation methods, are topics of interest. In these processes, temperature plays a pivotal role in influencing the formation of primary products and by-products, especially in direct pyrolysis techniques. Additionally, the feedstock types, reaction environment, and operating conditions in the pyrolysis process are still vital factors for the production and compositions of LBC. Compared to traditional pyrolysis methods, HTC methods offer advantages such as enriched surface functional groups and an enhanced calorific value. However, challenges like lower selectivity and the generation of potential by-products are also noteworthy. Addressing the limitations of high temperature and energy consumption in HTC methods, lowering the reaction temperature in LBC processes is optional. MAC, which has been successfully applied in the production of biochar and biofuels, is expected to become more economical with reductions in feedstock costs and improvements in equipment technology. TDC, which is able to balance pore structure and stability, is crucial for specific applications. However, its feasibility and effectiveness under different conditions and in various application domains remain subjects for further research and validation, and novel template methods can be a favorable choice. Additionally, the combination of physical and chemical activation and dual-chemical activation are promising methods for LBC with higher activation efficiency and more pore volume, making it extensively applicable in the preparation process. Moreover, the recent advances in the application of LBC are still focused on adsorption, catalysis, and energy storage, and applications in more fields need to be explored. This review provides a comprehensive comparative analysis of different preparation methods and applications of LBC in recent years, facilitating a deeper understanding of their advantages and limitations for researchers in sustainable applications. Sustainable and environmentally friendly alternatives are offered, which reduce reliance on traditional energy sources and mineral resources, thereby providing practical solutions to environmental and energy challenges.

## Figures and Tables

**Table 1:** Comparison of different carbonization methods of LBC.

Direct Carbonization		Hydrothermal Carbonization	Template-Directed Carbonization	Microwave-Assisted Carbonization
Slow Pyrolysis	Fast Pyrolysis			
Slow heating rates (0.02 °C/s to 1 °C/s) Long residence time (several hours to days) Low temperature (300–700 °C) Higher yields of LBC produced Limitations in long processing cycles and low energy efficiency	Rapid heating rates (>2 °C/s) Short residence time (<10 s) A wide temperature range (300–1000 °C) Efficient production of bio-oils Lower yields of LBC produced	Water acts as an efficient heat transfer medium The surfaces of LBC are produced with rich functional groups Enhanced calorific value Lower selectivity and the potential generation of unwanted by-products Less energy input to produce	The produced LBC has adjustable pore sizes and stable structures The templates used are relatively inexpensive and readily available More complex carbonization processes	Microwave acts as a main heating source Internal heating Reduced energy consumption and shorter processing times LBC obtained has more uniform chemical properties Need for microwave absorbers in the feedstock and hotspot phenomena

**Table 2:** Comparison of different activation methods of LBC.

Physical Activation	Chemical Activation	Physicochemical Activation
Oxidizing gases like water vapor, carbon dioxide, oxygen, air, or their mixtures as activating agents Simplicity and no secondary pollution Limitations in long activation time, high energy consumption, and low activated carbon yield	Higher activation efficiency, less carbon burn-off, and higher yield of activated carbon Relatively shorter operating time, lower operating temperature, and higher pore volume Chemical consumption, equipment corrosion, chemical recovery, and secondary pollution	Low-cost and abundant raw materials Activated carbon produced with excellent adsorption performance

## Data Availability

Not applicable.
